# Increased food supply mitigates ocean acidification effects on calcification but exacerbates effects on growth

**DOI:** 10.1038/s41598-018-28012-w

**Published:** 2018-06-28

**Authors:** Norah E. M. Brown, Joey R. Bernhardt, Kathryn M. Anderson, Christopher D. G. Harley

**Affiliations:** 1 School of Environmental Studies, University of Victoria, Victoria, BC Canada; 2 Department of Zoology, University of British Columbia, Vancouver, BC Canada; 3 School of Biological Sciences, Washington State University, Pullman, WA USA; 4 Institute for the Oceans and Fisheries, University of British Columbia, Vancouver, BC Canada

## Abstract

Because many of the negative effects of ocean acidification on marine life may result from underlying energetic short-falls associated with increased metabolic demands, several studies have hypothesized that negative responses to high CO_2_ could be reduced by energy input. Although this hypothesis was supported by a recent meta-analysis, we believe that the meta-analytic calculation used was not appropriate to test the stated hypothesis. Here, we first clarify the hypothesis put forward, the crux being that the effects of increased food supply and CO_2_ interact statistically. We then test this hypothesis by examining the available data in a more appropriate analytical framework. Using factorial meta-analysis, we confirm that food addition has a positive effect and CO_2_ has a negative effect on both growth and calcification. For calcification, food addition did indeed reduce CO_2_ impacts. Surprisingly, however, we found that food addition actually exacerbated the effects of acidification on growth, perhaps due to increased scope upon which CO_2_ effects can act in food-replete situations. These interactive effects were undetectable using a multilevel meta-analytic approach. Ongoing changes in food supply and carbonate chemistry, coupled with under-described, poorly understood, and potentially surprising interactive outcomes for these two variables, suggest that the role of food should remain a priority in ocean acidification research.

Arising from: L. Ramajo et al., Sci. Rep. 6: 19374 (2016).

## Introduction

Ocean acidification (OA) has been recognized as one of the most significant threats to marine life^1^, with demonstrated negative effects across many taxa in terms of growth and calcification^2^. In many cases, impairment by acidification reflects an underlying energetics problem, as organisms may need to redirect energy from growth and reproduction towards acid-base regulation, especially at sites of calcification^3,4^. Given that sub-lethal negative effects of OA can result from this energetic trade off, a number of studies have proposed the hypothesis that negative responses to OA could be minimized in situations where energy (e.g., food) is more readily available^5^. If true, this has far-reaching implications for how we both understand and study OA^6,7^. Although the energy limitation hypothesis is ecologically relevant and physiologically plausible, many of the recent tests of the idea suffer from a lack of adequately framed and precisely stated expectations regarding the combined effects of OA and food supply, or from inappropriate statistical comparisons among different experimental groups. Perhaps as a result of these framing of expectations, the most appropriate meta-analytical techniques have not yet been employed to seek generalities across studies. Here, We (1) clarify the hypothesis put forward by a recent meta-analysis (Ramajo *et al*.^5^) and others^8–19^, and (2) test the hypothesis using available data in a more appropriate meta-analytical framework.

**Figure 1:**
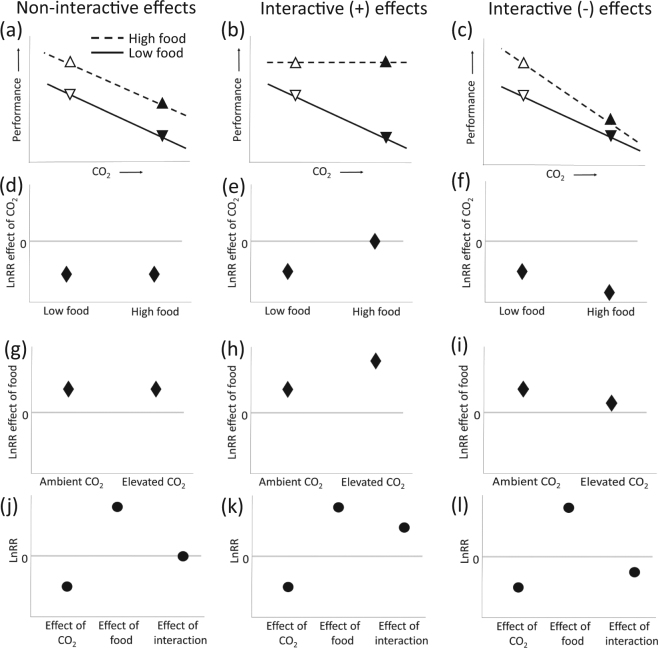
Schematic demonstrating hypothetical responses of organisms where high CO_2_ has a negative effect and high food supply has a positive effect on performance. Left-hand panels show a scenario where organismal response to high CO_2_ is not modified by food supply, demonstrating a non-interactive effect of food and OA on performance (**a**). Middle and right-hand panels depict interactive effects of food and ocean acidification on performance, where organismal response to high CO_2_ is modified by food supply in a positive (**b**) or negative (**c**) way. The outcomes of (**a**–**c**) could be realized across many studies in a meta-analysis, where the mean effect sizes (in Ln response ratio, LnRR) of OA may remain the same, regardless of food addition (**d**) or differ under food supplementation (**e**,**f**). In (**d**), while higher food may be beneficial and OA may be detrimental, a change in food supply does not alter the magnitude of the OA effect. Similarly, the effect of food supply could have no relationship with CO_2_ (**g**) or be influenced by change in CO_2_ (**h**,**i**). Finally, in a factorial meta-analysis, the interaction between food supply and OA could be non-significant (**a** simple multiplicative outcome, in this case (**j**), significantly positive (**k**), where the interaction food and OA on average improves the performance of organisms relative to the non-interactive case (compare b to a), or significantly negative (**l**), where the interaction between food and OA on average decreases performance of organisms relative to the non-interactive case (compare c to a).

The crux of the hypothesis that a negative response to OA by calcifiers could be modified by food availability lies in the idea that increased food supply does not simply offset some of the negative consequences of ocean acidification; rather, it implies a positive *interactive* effect of food and OA (*sensu* Morris *et al*.^20^). For this type of statistical interaction, the term ‘positive’ refers to the fact that the observed response to simultaneously elevated food and CO_2_ is more positive, or less negative, than the response predicted from the single-factor effects by a null, non-interactive model. The proper statistical test of the food limitation hypothesis would therefore be to estimate the *statistical* interaction between food supply and ocean acidification where the effect of OA on performance is stronger with limited food and weaker with abundant food. The distinction between this positive interactive scenario and two alternatives (non-interactive and negative interactive scenarios) is outlined in Fig. 1. The non-interactive scenario is one in which the effect of OA does not depend on food availability (Fig. 1a, slopes are equal). Here, we use the term ‘non-interactive’ to imply a null additive model in multilevel meta-analysis and a null multiplicative model in factorial meta-analysis. In the positive interactive scenario, the response of an organism to acidification is altered by food addition such that the negative effect of OA apparent under low food conditions is reduced or disappears when food supply is high (Fig. 1b, low food slope is more negative). Finally, in the negative interactive scenario, food addition actually promotes a stronger negative response to CO_2_ (Fig. 1c, high food slope is more negative). This last case is not widely predicted, but thus far the only example of a significant interaction between factorially manipulated CO_2_ and food supply is of this type (see Cole *et al*.^21^). These three hypotheses lead to how food supply alters the effect of OA (Fig. 1d–f) and, equivalently, how OA affects an animal’s response to food supplementation (Fig. 1g–i). Using a factorial meta-analysis, we can further test if a negative effect of CO_2_ and a positive effect of food supply can combine in a multiplicative way (i.e. no interaction, Fig. 1j) or in an interactive way, either where the interaction on average improves (Fig. 1k) or decreases performance relative to the non-interactive null expectation (Fig. 1l).

Ramajo *et al*.^5^ set out to explore these possibilities using 12 studies that factorially manipulated OA and food supply. However, rather than comparing OA effects at high and low food supply (i.e. slopes of lines, Fig. 1a–c), Ramajo and colleagues compared both the high food acidified treatment (solid upward-pointing triangles, Fig. 1a,b) and the low food acidified treatment (solid downward-pointing triangles, Fig. 1a–c) to a single reference treatment: high food, non-acidified (open upward-pointing triangles, Fig. 1a–c). This comparison using a common denominator is atypical in the meta-analysis literature, where, in subgroup analyses, independent effect sizes are computed within subgroups^22^. Because the effect size of acidification was not calculated independently for each subgroup (level of food supply) by Ramajo *et al*.^5^, the response ratios (lnRR) presented for low and intermediate food supply cannot be considered true effect sizes and thus cannot be used to compare if the effect of CO_2_ changes under food supply levels. Therefore, the authors inadvertently tested a substantially different hypothesis, namely that when food is abundant, increasing CO_2_ has a small effect, but that *simultaneous* exposure to OA *and* food deprivation has a large negative impact relative to well-fed, non-acidified controls. Because the analysis did not include any data from organisms under ambient CO_2_ conditions and low or intermediate food supply (open down-facing triangles, Fig. 1a–c), the analysis cannot determine if or how the response to OA changes with food supply (i.e. cannot distinguish between the interactive vs. non-interactive scenarios outlined in Fig. 1).

To clarify the relationship between OA effects and food supply, we re-examined the available literature using two different types of meta-analysis: multilevel meta-analysis (employed by Ramajo *et al*.^5^ and others^23^ and factorial meta-analysis^20,24,25^. Within each of these types of meta-analysis we used three different datasets to test the hypothesis that the response to OA is modified by food supply. We first created an updated dataset by extracting data from the eleven published studies in Ramajo *et al*.^5^ and six additional studies^11,19,21,26–28^ (see Supplementary Information, Table S1). In order to compare these results to findings in Ramajo *et al*.^5^, we next replicated their dataset twice, first including the incorrect log response ratio (LnRR) calculation along with other errors in the dataset, and secondly by correcting the LnRR calculation in order to demonstrate the impact that this miscalculation makes on the results and inferences (see Supplementary Information).

## Results and Discussion

**Figure 2:**
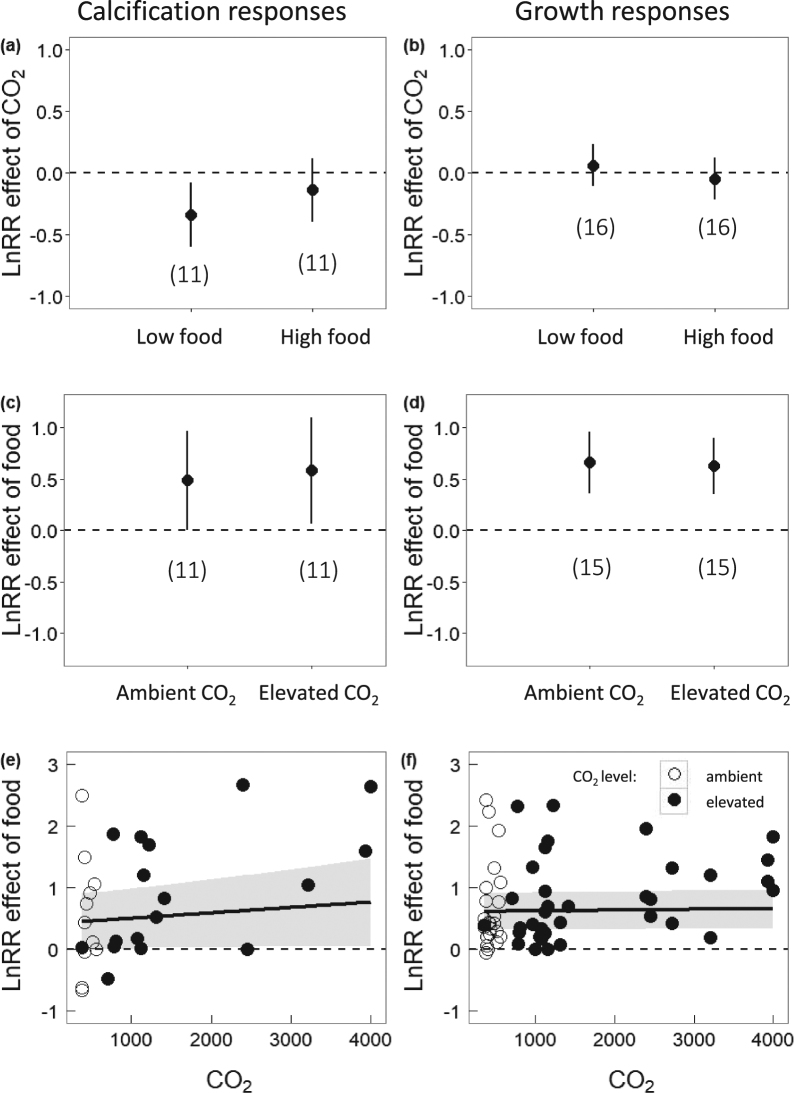
Mean effect sizes (LnRR) for the responses of calcification (left hand panels) and growth (right hand panels) to food supply and elevated CO_2_. Mean responses to elevated CO_2_ did not depend on food supply for (**a**) calcification (Q_M(coef)_ = 1.84, *P* = 0.17) nor (**b**) growth (Q_M(coef)_ = 1.31, *P* = 0.25). Across food treatments, there was a significant negative response to high CO_2_ for (**a**) calcification (Q_M_ = 6.56, *P* = 0.037) but not (**b**) growth (Q_M_ = 1.33, *P* = 0.52). Response to high food was not different between elevated and ambient CO_2_ levels for (**c**) calcification (Q_M(coef)_ = 2.62, *P* = 0.11) or (**d**) growth (Q_M(coef)_ = 1.64, *P* = 0.20) responses, nor did response to high food supply depend on continuous CO_2_ for (**e**) calcification (Q_Mslope_ = 1.72, *P* = 0.19, slope = 0.0001) or (**f**) growth (Q_Mslope_ = 0.48, *P* = 0.49, slope = 0.00). Across OA levels, food had no effect (but trending positive) on (**c**) calcification (Q_M_ = 5.33, *P* = 0.069) and a significant positive effect on (**d**) growth (Q_M_ = 20.59, *P* < 0.0001). Across a continuous CO_2_ gradient, food had a significant positive effect on (f) growth (Q_M(intercept)_ = 15.74, *P* < 0.0001), but not (**e**) calcification (Q_M(intercept)_ = 3.19, *P* = 0.074) responses. Parameter estimates and confidence intervals from fitted REML models. Symbols represent the treatment in each study from which the LnRR was calculated (open vs. closed circles for ambient and elevated CO_2_, respectively). Numbers in brackets indicate the number of studies contributing to the LnRR; for (**e**) and (**f**), n = 11 and n = 15, respectively.

**Figure 3:**
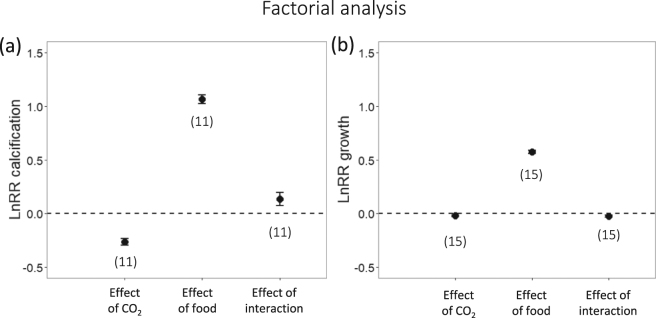
Factorial analysis. Overall and interactive effect sizes for CO_2_ and food supply for (**a**) calcification and (**b**) growth responses. (**a**) Here, we found that overall, CO_2_ had a significant negative effect on calcification (LnRR = −0.26, lower = −0.29, upper = −0.23), whereas food addition had an overall significant positive effect on calcification (LnRR = 1.07, lower = 1.03, upper = 1.11). The interaction between CO_2_ and food supply was significantly positive for calcification (LnRR = 0.13, lower = 0.07, upper = 0.19), meaning that OA effects were weaker (less negative) at high food. (**b**) For growth responses, CO_2_ had a small but significant negative effect (LnRR = −0.020, lower = −0.026, upper = −0.013), food had a positive effect (LnRR = 0.57, lower = 0.56, upper = 0.59). The interaction between CO_2_ and food supply was small but significantly negative for growth (LnRR = −0.024, lower = −0.035, upper = −0.012), meaning that OA effects were stronger (more negative) at high food. Error bars are 95% confidence intervals calculated from standard error for small sample sizes. If the error bars do not overlap zero then a significant response is inferred. Numbers in brackets indicate the number of studies contributing to the LnRR.

Using the updated dataset, we calculated log-response ratio (LnRR) of the effect of CO_2_ using response to OA/control (solid vs. open symbols, Fig. 1a,b) where control was low CO_2_ conditions under the same food supply level as the OA treatment. We found non-interactive effects of OA and food supply for both growth and calcification. In other words, the response to OA did not depend on food supply (calcification Q_M(coef)_ = 1.84, *P* = 0.17, Fig. 2a; growth Q_M(coef)_ = 1.31, *P* = 0.25, Fig. 2b). Furthermore, restricting this analysis to the data and methods used by Ramajo *et al*.^5^ did not alter our conclusions (calcification Q_M(coef)_ = 0.60, *P* = 0.44, Fig. 3b, and growth Q_M(coef)_ = 0.28, *P* = 0.61, Fig. S2d). With regard to the data plotted in Figs 2a and 3b, we remind readers that a statistical difference cannot be inferred between a significant result and a non-significant result^29^, although it is a common mistake to draw this conclusion when the confidence interval crosses zero in one case but not the other. In other words, a difference in statistics does not equate to a statistical difference.

Across food treatments, we detected an overall significant negative response to high CO_2_ for calcification-related responses (Q_M_ = 6.56, *P* = 0.037, Fig. 2a). For calcification, the effect size of OA was positively related to the experimental change in CO_2_ (Q_M_ = 10.79, *P* = 0.0045, Fig. S1a). In contrast, overall growth responses to OA were less variable, did not differ from zero (Q_M_ = 1.33, *P* = 0.52, Fig. 2b), and did not change with increasing experimental change in CO_2_ (Q_M_ = 2.37, *P* = 0.30 Fig. S1b). These results concur with a recent meta-analysis showing that calcification responses to OA across many taxa were stronger than growth responses^2^. The non-significant growth response to OA found here suggest either that our analysis represents an insufficient sample size to detect a true effect or that the species tested were relatively resistant to acidification compared to responses reported elsewhere^2^.

Across CO_2_ levels, food supply had no effect (but trending positive) on calcification (OA: Q_M_ = 5.33, *P* = 0.069, Fig. 2c; continuous CO_2_: Q_M(intercept)_ = 3.19, *P* = 0.074, Fig. 2e) and a strong positive effect on growth (OA: Q_M_ = 20.59, *P* < 0.0001, Fig. 2d; continuous CO_2_: Q_M(intercept)_ = 15.74, *P* < 0.0001, Fig. 2f). The response to high food supply did not depend on OA manipulation or continuous CO_2_ level for growth (OA: Q_M(coef)_ = 1.64, *P* = 0.20, Fig. 2d; continuous CO_2_: Q_M_ = 0.48, *P* = 0.49, Fig. 2f) or calcification responses (OA: Q_M(coef)_ = 2.62, *P* = 0.11, Fig. 2c; continuous CO_2_: Q_M_ = 1.73, *P* = 0.19, Fig. 2e). These results suggest that growth may be more tightly linked to food supply than OA, while OA may be relatively more important for calcification.

As an alternative approach, we next employed a factorial meta-analysis on the same data to calculate overall effects of CO_2_ and food addition and their interaction. This analysis is a powerful tool when using fully factorial datasets (i.e. each experiment manipulated both OA and food supply)^20,24,25^. Here, we confirm that CO_2_ has a significant negative effect on calcification (LnRR = −0.26, lower = −0.29, upper = −0.23, Fig. 3a), as found in Fig. 2a. For growth in this analysis we were able to detect a small, significant negative effect of CO_2_ (LnRR = −0.020, lower = −0.026, upper = −0.013, Fig. 3), whereas we were unable to detect this effect using multilevel meta-analysis (Fig. 2b). In this analysis, we found strong significant overall effects of food on both calcification (LnRR = 1.07, lower = 1.03, upper = 1.11, Fig. 3a) and growth (LnRR = 0.57, lower = 0.56, upper = 0.59, Fig. S2b), whereas previously we found a significant effect on growth only and a positive trend for calcification (Fig. 2c–f). When analyzed factorially, we found that the interaction between OA and food supply had a small and significant positive effect on calcification (LnRR = 0.13, lower = 0.07, upper = 0.19, Fig. 3a). This result provides support for the positive interaction hypothesis that food addition can ameliorate response to CO_2_ (Fig. 1b). For growth, we found support for the opposite hypothesis, where the interaction between food supply and CO_2_ had a small but significant negative interactive effect on growth (LnRR = −0.024, lower = −0.035, upper = −0.012, Fig. 3b). This result implies that negative CO_2_ effects are actually more severe at high food concentrations, perhaps because high food conditions support a larger scope for the effect of CO_2_ to act on (Fig. 1c).

When we restricted the factorial analysis to the data used by Ramajo *et al*.^5^, we also found negative effects of CO_2_ and positive effects of food on both calcification and growth responses. In addition, the interactive effect of food supply and OA on calcification was of similar magnitude (LnRR = 0.10, lower = 0.043, upper = 0.15, Fig. S3b). However, using only data from Ramajo *et al*.^5^, we found that the interactive effect of CO_2_ and food supply on growth was slightly positive (LnRR = 0.03, lower = 0.015, upper = 0.048, Fig. S3d), which is in the opposite direction to the same analysis using more data (Fig. 3b). However, a change in sign of the interactive effect is an indication of extraction errors in the original Ramajo *et al*.^5^ dataset (Table S1), as re-analysis without extraction errors yields a non-interactive effect. Therefore, the negative interaction effect on growth is only detectable with a larger sample size.

Overall, there was no consistent tendency for the effects of OA on growth and calcification to be modified in the same way by food supply. We found that for calcification there is evidence that positive effects of food supply do mediate negative response to acidification. For growth responses, in contrast, we found a significant negative interactive effect using factorial analysis, and no response using multilevel meta-analysis. Although both statistically sound methods, factorial analysis likely has more power to accurately estimate the effect size of the interaction and is particularly useful for understanding the growing list of multiple stressor studies^30,31^. The findings of the multilevel analyses align more with the findings of the individual studies making up the meta-analysis, where the interaction between food and OA was non-significant in 21 of 22 cases, although, it should be noted that the single significant interactive case revealed an increased OA effect on growth at higher food rather than a decreased one^21^ (consistent with Fig. 1c and findings of factorial meta-analysis on growth). The number of studies considered here and by Ramajo *et al*.^5^ is relatively low, although neither sensitivity nor bias analyses indicated cause for concern (see methods for details). It is also worth noting that some of the included studies did find interactive effects for other response variables where negative responses to OA were ameliorated under high food, including metabolic rate^17^, aragonite crystal length^15^ and, frequency of normal development^21^, suggesting food supply could be important in mediating other physiological and developmental responses to OA beyond calcification and growth responses.

There may be physiological mechanisms, yet to be explored, for the various hypotheses described above. Mechanistically, for added food to reduce the negative impact of OA (i.e., a positive interactive outcome), either feeding rates would need to increase to compensate for higher energetic demands, or energy acquisition from ingested food would need to be more efficient. The reverse would need to be true to support the negative interactive outcome, where OA impacts are most severe at high food supply. Currently, there is little evidence to support compensatory feeding under acidification. In fact, feeding and clearance rates have been shown to decline under acidification across a wide range of taxa, both herbivorous and carnivorous, encompassing a variety of feeding appendages and behaviours^32^, which may help to explain the negative interactive effect we found for growth. Similarly, available evidence does not suggest that increased assimilation efficiency is likely to be common under high CO_2_^33–35^. From a physiological standpoint, therefore, the mechanisms underlying the significant positive interactive effects on calcification described here are yet to be pinpointed. It should be noted, however, that reductions in search and handling times and increases in palatability under acidification^36,37^ could influence an organism’s energetic balance in such a way that OA costs are minimized by higher food availability, but most published experiments – including those in our meta-analysis – do not allow for these types of longer-term effects to emerge. Finally, it is important to recognize that food supply could mediate OA effects in a non-interactive scenario for one response variable (i.e. maximum metabolic rate), but appear interactive for another variable if the combined effects of OA and food limitation crosses some important physiological threshold (e.g., metabolic supply becomes insufficient to meet demand and the animal dies) or ecological threshold (e.g., population intrinsic growth rate, λ, drops below 1)^38^. To the best of our knowledge, these scenarios have not been explicitly tested.

Moving forward, we recommend that researchers take great care when framing and testing hypotheses related to the effects of food availability on responses to OA. The individual studies in this meta-analysis overwhelmingly found non-interactive effects, yet several authors use phrasing such as “food supply reduces the impacts of experimental OA”^5^, “failure to provide food can increase vulnerability to OA in experimental assessments”^5^, “food supply… can mitigate the negative impacts of future OA”^12^, “feeding and energy availability can mediate reductions in growth due to OA stress”^18^, and “Zooplanktivory ameliorates the effects of ocean acidification”^8^. Others have pointed out this discrepancy, for example Drenkard *et al*.^16^ comment on the misclassification of non-interactive results as interactive by Edmunds^8^ and underscore that, despite misinterpretation, both of their datasets confirm that energetic status does not alter calcification sensitivity to ocean acidification. The authors of the above papers may have intended to convey that the negative effects of OA at low food availability can be *offset* by supplying more food. We believe the language used above could equally be interpreted as a modification of the effect of OA (i.e. an interactive effect) or an offsetting of the effect of OA (i.e. a non-interactive effect). Careful and deliberate consideration of language used to describe experimental results can help guide further mechanistic experiments to enhance our understanding of ocean acidification responses.

Many of the studies cited here were driven by observations in the field where ecosystems are observed to be healthy in the presence of high food despite acidic conditions^9,12,39^. In the absence of a non-acidic, high food ‘control’, however, it isn’t clear if acidic conditions are truly having no effect, or if there is simply no difference between non-acidified, low food situations and acidified, high food situations. Of course, this does not rule out the broader contention that adding more food will allow organisms to maintain performance under increasing acidification; indeed, that outcome would be expected from a non-interactive scenario (compare the open downward-facing triangle to the filled upward-facing triangle in Fig. 1a). However, if this argument is applied to global change scenarios generally, it would be akin to comparing a present-day ocean with little food to a future ocean with an abundance of food. While it is certainly possible that OA can increase food availability (e.g., for some herbivores^7^), this will not be the case for all species, nor in all habitats, and the opposite can also be true^40^. Unless changes in food supply through time are known in advance, the proper comparison is the effect of OA (elevated vs. ambient CO_2_) at low food supply compared to the effect of OA (elevated vs. ambient CO_2_) at high food supply (Fig. 1a–c solid and dashed lines, respectively). Overall, our results suggest that the ultimate role of food as a modifying influence on ecological responses to ocean acidification will depend on the relative importance of calcification and growth to fitness and population, community, and ecosystem dynamics. Further, we suggest that future changes in ocean acidity could affect systems of both high and low productivity and food availability, but the ecological effects may be difficult to predict without knowledge of the ways in which acidification indirectly affects food supply.

Understanding the distinction between interactive and non-interactive effects is important for furthering our mechanistic understanding of ocean acidification. If these effects are indeed non-interactive (as suggested by multilevel meta-analysis), we can make specific predictions about the effect of OA separately from the effect food, allowing us to have somewhat more confidence in cautiously predicting OA effects for food supply conditions not directly considered in the original studies. However, the negative interactive effect on growth (found in the factorial analysis) suggests that ecological contexts in which food is super-abundant may be more susceptible to OA effects than more food-limited systems, and the converse is true in terms of calcification. The presence of mixed interactive effects prevents us from generalizing broadly from a limited set of results as the outcome of one effect cannot be predicted independently from the other and suggests that future OA impacts may vary meaningfully from one context to another.

## Conclusions

Overall, we provide evidence that food supply both mitigates and worsens the negative effects of OA, depending on the response variable. Clearly, there remains much work to be done on this topic. We agree with Ramajo *et al*.^5^ that researchers should, where possible, explicitly relate food supply levels to natural conditions (e.g.^9,16^) and organismal requirements, as interactive effects of food supply and OA could emerge when crossing ecological thresholds. Furthermore, if and when “low food” treatment levels equate to starvation of the organism, researchers should consider that other energetic processes, such as metabolic depression, are likely to become important and this could change the response to OA. More studies are needed to determine the full extent to which responses to OA change across a wide range of food supply levels and feeding modes. The careful selection of natural field-relevant food supply levels can help identify when responses to OA are likely to cross ecological thresholds, which can generate interactive outcomes at higher levels of biological organization even when the underlying effects are non-interactive. Given that both food supply and marine carbonate chemistry are changing simultaneously through time, a more detailed understanding of the inter-relationship of their effects, both physiological and ecological, remains a research priority.

## Methods

Two meta-analytic techniques were explored to test the hypotheses posed above. The first, multilevel meta-analysis, uses ln response ratios of the effect of CO_2_ (comparing across food levels) and the effect of food (comparing across CO_2_ levels). The second technique, factorial meta-analysis, is suitable for papers that employ fully-factorial experiments. The analysis used by Ramajo *et al*.^5^, specifically their calculation of effect size, was not appropriate for the stated hypothesis and, this mismatch calls their conclusion into question. We have therefore replicated results from Ramajo *et al*.^5^, using both types of meta-analysis. These methods can be found in the Supplementary Information.

### Multilevel meta-analysis

We extracted data on growth and calcification responses to CO_2_ from 17 articles in which both food supply and CO_2_ were manipulated. (Figure 2) All data (68 observations for calcification measures, 144 for growth measures) were extracted from figures using WebPlotDigitizer^41^. The papers used in the multilevel analysis were all the published articles from Ramajo *et al*.^5^ and six additional articles found during an ISI Web of Knowledge search using similar search terms (Supplementary Information, Table S1). Here, we used the width variable from Taylor *et al*.^26^ because this is the most commonly used measure of urchin growth in both fisheries and population-level literature. Furthermore, Taylor *et al*.^26^ was only used for the LnRR effect of CO_2_ analysis but not for the effect of food analysis since negative and positive values cannot be compared using LnRR. We only used the aggregate ambient CO_2_ level described in Maier *et al*.^19^ and only one elevated CO_2_ level (intermediate) because, as above, LnRR cannot evaluate dissolution at the same time as calcification. We added a constant (0.5%) to all percentage growth measurements in Büscher *et al*.^11^ since values close to zero approach infinity when taking ln(0).

Meta-analytic calculations and statistics were performed in R (Version 3.3.1, R Development Core Team^42^), using the *metafor* package^43^. We calculated a weighted ln Response Ratio (lnRR) using OA/Control within each food supply level (*escalc* function, *metafor* package in R^42,43^). We constructed multilevel models with food supply as a fixed effect and aspects of non-independence as random effects using the *rma*.*mv* function in R^43^ and checked these models for publication bias (using a contour-enhanced funnel plot^44^) and sensitivity to outliers (following methods in Habbeck *et al*.^45^). We used restricted maximum likelihood (REML) approaches to test if effect size estimates were significantly different than zero and significantly different between food supply and CO_2_ levels. Some levels of CO_2_ were different between food levels within an experimental treatment, therefore we averaged between food levels to obtain a single CO_2_ level for each low food-high food pair to regress against the LnRR effect of food. We excluded intermediate food levels because the sample size was too small to make meaningful comparisons. The data used included several non-independent measures: common control for multiple levels of CO_2_, multiple variables for same response (growth responses only), and multiple independent studies within the same article. We accounted for dependent sampling errors caused by using a common control for multiple levels of CO_2_ by constructing a variance-covariance matrix of the effect size estimates^46^. We accounted for multiple variables for the same response and multiple independent studies within an article by including an unstructured random effect of response variable unit (of the form ~Unit|Paper_no) and food supply (of the form ~Food.supply|Paper_no) which allows the random effects to have different variances for each outcome while also allowing random effects to be correlated^47^. We plotted estimates for the mean and confidence intervals generated from the rma.mv models. For the calculated LnRR of food supply (high food/control, low food), we used data from all CO_2_ levels, therefore there was an unequal number of observations in each category. To account for this, we included an unstructured random effect for CO_2_ level (of the form ~ CO_2__level|Paper_no) in addition to an unstructured random effect of response variable unit (of the form ~Unit|Paper_no). We tested all models first for an overall effect of the moderator (Q_M_), food supply or CO_2_, and then tested between levels of the moderator – i.e. testing for the interaction between food and CO_2_ (Q_Mcoef_), e.g., High vs. Low food or Ambient vs. Elevated CO_2_. These tests of heterogeneity described by the moderators (Q_M_ tests) are akin to a Wald-type Chi-squared test^43^. When there was only a single moderator (as in Fig. 2e,f), the effect of the moderator (e.g., CO_2_) is a test of the slope vs. zero (Q_Mslope_) and the overall response (regardless of moderator) is a test of the intercept vs. zero (Q_Mintercept_).

### Factorial Meta-analysis

For this analysis, we used the same data as the multilevel analysis (Fig. 2), with one exception: Taylor *et al*.^26^ was excluded because we cannot take the ln of negative values (as above). (Figure 3) In order to account for multiple CO_2_ levels per paper, we averaged effect sizes across a single paper, as factorial LnRR are not appropriate for constructing a variance-covariance matrix to account for multiple comparisons to the same CO_2_ control. We accounted for multiple responses per paper in this analysis in the same way. Multiple study sites within papers were considered to be independent enough to consider separately.

We calculated ln response ratios for overall and interactive effects of food limitation and high CO_2_, using factorial meta-analysis methods^20,24,25^. Overall effects incorporate the effects of the interacting factor at all levels. In this analysis, we assume effects (food supply and high CO_2_) are multiplicative, which is a more biologically realistic null model than simply additive (i.e. “you can’t die twice”^48^). We calculated overall and interactive effect sizes and sampling variance from equations Appendix B.7 to B.12 in Morris *et al*.^20^. We weighted effect sizes by inverse sampling variance using a random effects model where weights were assigned using within study variance plus between studies variance^49^ and corrected for the small number of studies using equation 7 in Hedges *et al*.^50^. We constructed 95% confidence intervals using equation 8 in Hedges *et al*.^50^ and inferred significance from these confidence intervals. If the interaction effect is not different than zero (CIs overlap zero), then we can infer multiplicative effects^20^. If otherwise, we infer significant interactive effects: if the interaction effect is positive (above zero), then the interaction between the two factors has a positive effect on performance relative to the simple multiplicative case; if negative (below zero), we infer the interaction of the two factors has a negative effect on performance relative to the multiplicative scenario^20,51–55^.

## Electronic supplementary material

Supplement
