# Author Correction: Increased food supply mitigates ocean acidification effects on calcification but exacerbates effects on growth

**DOI:** 10.1038/s41598-018-28761-8

**Published:** 2018-07-10

**Authors:** Norah E. M. Brown, Joey R. Bernhardt, Kathryn M. Anderson, Christopher D. G. Harley

**Affiliations:** 1 School of Environmental Studies, University of Victoria, Victoria, BC Canada; 2 Department of Zoology, University of British Columbia, Vancouver, BC Canada; 3 School of Biological Sciences, Washington State University, Pullman, WA USA; 4 Institute for the Oceans and Fisheries, University of British Columbia, Vancouver, BC Canada

Correction to: *Scientific Reports* 10.1038/s41598-018-28012-w, published online 28 June 2018

In the original version of this Article, Figure 3 was a duplication of Supplementary Figure S2. This has now been corrected in the HTML and PDF versions of this Article.

