# Supplementary material for: Increased food supply mitigates ocean acidification effects on calcification but exacerbates effects on growth
Source: Sci Rep. 2018 Jun 28;8:9800. doi: 10.1038/s41598-018-28012-w (PMC6023940; doi:10.1038/s41598-018-28012-w)
Supplement: Supplementary file 1 — Supplement [file 41598_2018_28012_MOESM1_ESM.docx]

Supplementary Information

Increased food supply mitigates ocean acidification effects on calcification but exacerbates effects on growth

Norah E. M. Brown*^1^, Joey R. Bernhardt^2^, Kathryn M. Anderson^3^, and Christopher D. G. Harley^2,4^

^1^ School of Environmental Studies, University of Victoria, Victoria, BC, Canada

^2^ Department of Zoology, University of British Columbia, Vancouver, BC, Canada

^3^ School of Biological Sciences, Washington State University, Pullman, WA, USA

^4^ Institute for the Oceans and Fisheries, University of British Columbia, Vancouver, BC, Canada

*Correspondence to norahbrown@uvic.ca

*Regression with delta CO_2_* *analysis* (Fig. S1)

We tested for differences in response to CO_2_ (slopes) across experimentally imposed changes in CO_2_ between food supply levels using Q_M_ tests (here the test statistic is referred to as Q_Mslopes_). Models included deltaCO_2_ as a co-variate and were tested as in the multilevel analysis (Fig. 2).

*Repeatability analysis* (Fig. S2)

Here we used exactly the same papers as the original analysis and included intermediate food supply levels. When possible, we used data extracted by Ramajo *et al.* (2016)*,* courtesy of Laura Ramajo, and then extracted the additional data points for “low food, low pH” ourselves (but see Table S1). We followed statistical methods as presented in Ramajo *et al.* (2016). We present the data as bias-corrected bootstrapped confidence intervals and plotted these on top of bias-corrected means (BCa method). We used tests of heterogeneity described by the moderators (Q_M_ tests) on a random-effects model (study as random) to test differences between food supply levels for effect sizes. We inferred the Q_M_ method from their results although it is not described their methods. We first recreated the Ramajo *et al.* analysis, using a common denominator of “Control CO_2_, High food” treatment for all LnRR calculations (i.e. at every food level this was the common denominator) (Fig. 3 left hand panels). We then used the same data but the proper LnRR calculation (Fig. 3 right hand panels). This repeatability analysis contains a few known errors (see Table S1), so should be interpreted only as a comparison of the two LnRR techniques, not as a reliable result.

*Repeatability analysis using factorial meta-analysis* (Fig. S3)

Here, we calculated the factorial LnRR using the same methods as above for Fig. 3, using data from the repeatability analysis, Fig. S2. We conducted both the repeated analysis with a LnRR calculation error and a repeated analysis with this error corrected. Although factorial meta-analysis uses a different calculation than the analyses in the main paper, we assumed the same common denominator of “Control CO_2_, High food” treatment employed by Ramajo *et al.* (2016) as the control in the factorial LnRR calculations. This analysis is for illustration purposes only as Ramajo *et al.* (2016) did not employ this analysis in their paper. The results of this analysis demonstrate how the interaction effect is exaggerated when LnRR is calculated using a common control.


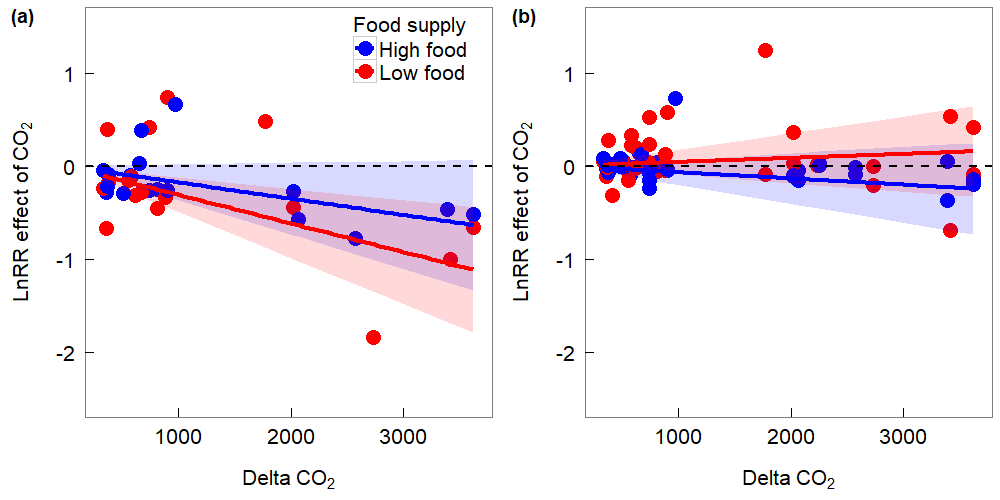


**Fig. S1.** Relationship between magnitude of CO_2_ effect imposed in the study on LnRR of (a) calcification (N=11) and (b) growth (N=16) measures under high (blue lines) and low food supply (red lines) conditions. The regression line and confidence intervals are predicted estimates and confidence intervals based on an REML model. Across food supply levels, calcification response ratios declined with increasing experimental change in CO_2_ (Q_M_ = 10.79, *P*=0.0045, slope high food = -0.003, slope low food = - 0.002). However, the rate of change of calcification LnRR with increasing delta CO_2_ (i.e. slope) did not differ between food treatments (Q_Mslopes_= 1.51, *P*= 0.22). Growth LnRR did not change with increasing experimental delta CO_2_ (Q_M_= 2.37, *P*=0.30, slope high food = -0.0001, slope low food = 0.00) and there was no difference in slopes between food treatments (Q_Mslopes_= 2.35, *P*= 0.12).

**
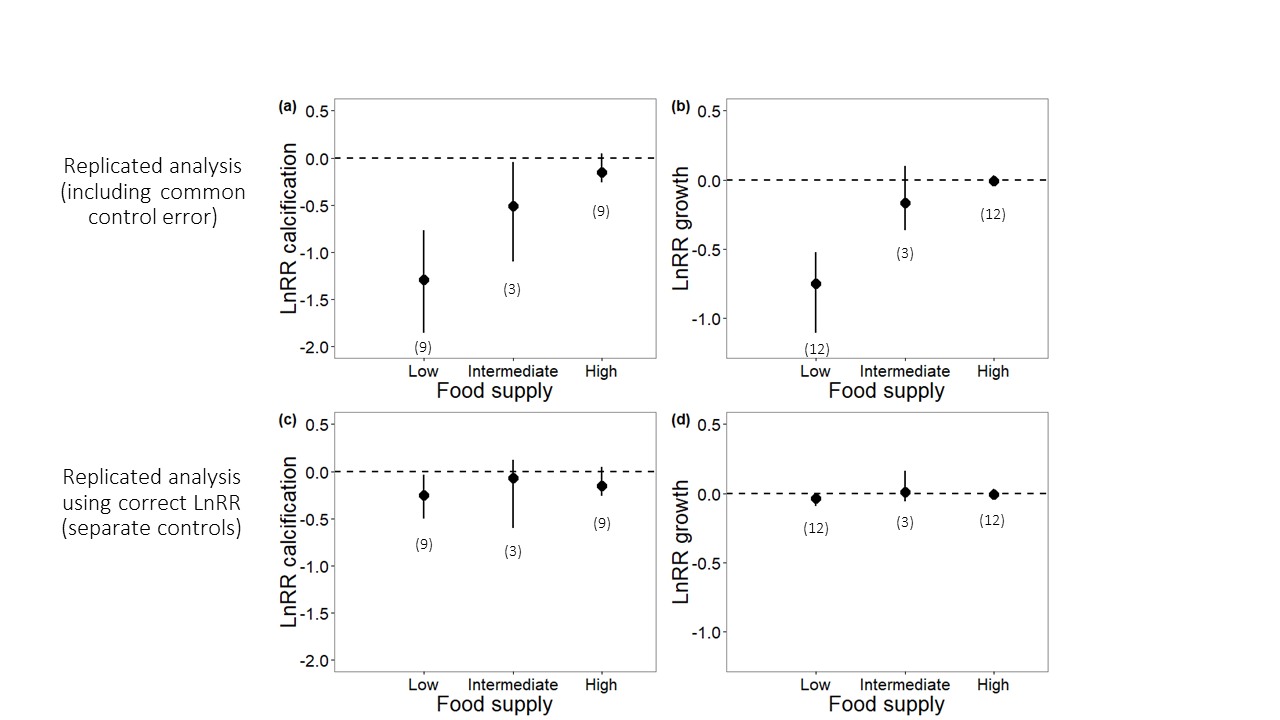
**

**Fig. S2.** Repeatability analysis. Mean effect size (LnRR) of (a,b) calcification and (c,d) growth responses to CO_2_ under low, intermediate, and high food supply levels using exact data from Ramajo *et al.* (2016) (see table S1). (a) and (b) show the analysis using the incorrect LnRR calculation employed by Ramajo *et al.* (2016) and (c) and (d) show how a corrected LnRR dramatically alters the results. Error bars shown are the bias-corrected bootstrapped 95% confidence intervals around bias-corrected estimates. In this analysis, if the 95% confidence intervals do not overlap between food supply levels a significant difference in CO_2_ response is inferred between those food supply levels. Therefore, the incorrect analysis (a) and (b) suggests that the response to CO_2_ is significantly different between low and high food supply for calcification (Q_M(coef)_=5.56, *P*= 0.018) and growth (Q_M(coef)_=6.45, *P*= 0.011); whereas the correct analysis (c) and (d) shows that the response to CO_2_ is not significantly different between food supply levels for calcification (Q_M(coef)_=0.60, *P*= 0.44) and growth (Q_M(coef)_=0.28, *P*= 0.61). Numbers in brackets indicate the number of studies contributing to the LnRR.


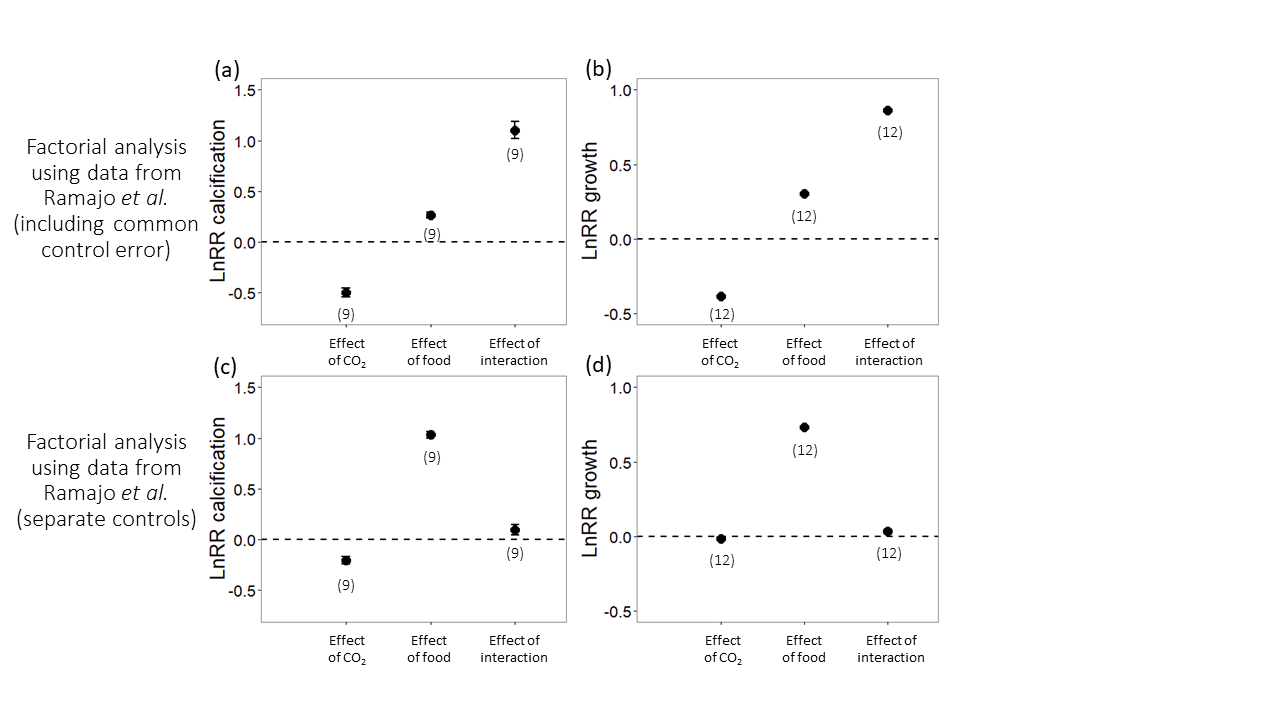


**Fig. S3.** Factorial repeatability analysis. Overall and interactive effect sizes for CO_2_ and food supply for (a,c) calcification and (b,d) growth responses using exact data from Ramajo *et al.* (2016)(see table S1). (a) and (b) show the analysis using the incorrect LnRR calculation employed by Ramajo *et al.* and (c) and (d) show how a corrected LnRR dramatically alters the results. (a) For calcification, the incorrect analysis shows an overall significant negative effect of CO_2_ (LnRR=-0.50, lower=-0.54, upper=-0.45), a significant positive effect of food supply (LnRR=0.26, lower=0.24, upper=0.29), and a very large positive interaction effect of CO_2_ and food supply (LnRR=1.10, lower=1.02, upper=1.19). (c) The corrected analysis shows a small significant negative effect of CO_2_ (LnRR=-0.20, lower=-0.24, upper=-0.17), a large significant positive effect of food supply (LnRR=1.03, lower=1.00, upper=1.07), and a much smaller positive effect of the interaction between CO_2_ and food supply (LnRR=0.10, lower=0.043, upper=0.15). (b) For growth, the incorrect analysis shows an overall significant negative effect of CO_2_ (LnRR=-0.39, lower=-0.39, upper=-0.38), a significant positive effect of food supply (LnRR=0.30, lower=0.29, upper=0.31), and a large positive interaction effect of CO_2_ and food supply (LnRR=0.86, lower=0.85, upper=0.87). (d) The corrected growth analysis shows a small significant negative effect of CO_2_ (LnRR=-0.014, lower=-0.024, upper=-0.0044), a large significant positive effect of food supply (LnRR=0.73, lower=0.72, upper=0.74), and a small positive effect of the interaction between CO_2_ and food supply (LnRR=0.03, lower=0.015, upper=0.048). We note that this interaction is in the opposite direction as calculated with more data in Fig. 2, however when calculated without extraction errors (Table 1) this effect becomes non-interactive. In both calcification and growth responses, the incorrect common control analysis inflated the interaction effect. Error bars are 95% confidence intervals calculated from standard error for small sample sizes. If the error bars do not overlap zero then a significant response is inferred. Numbers in brackets indicate the number of studies contributing to the LnRR.

**Table S1.** Differences between datasets used in Ramajo *et al*. (2016), the repeatability analysis, factorial analyses, and main analysis in Chapter 4.

|  | ***Ramajo et al. original analysis, Repeated analysis, Factorial analysis using Ramajo et al. data*** | ***Main analysis*** | ***Factorial main analysis*** |
| --- | --- | --- | --- |
| ***Calcification*** |  |  |  |
| *Hettinger et al. 2013* | total dry weight | shell growth | shell growth |
| *Edmunds 2011* | Calcification  mg/cm^2^/day | Calcification  mg/mg/day | Calcification  mg/mg/day |
| *Comeau et al. 2013* | Reversed feeding level for high CO_2_ for calcification measure (incorrectly used 0.22 mg CaCO_3_ day^-1^ mg^-1^ instead of 0.11) | Used correct high and low CO_2_ for calcification measure | Used correct high and low CO_2_ for calcification measure |
| *Edmunds 2011 &*  *Thomsen et al. 2013* | Used SE incorrectly as SD, artificially reducing variability | Used correct value for SD | Used correct value for SD |
| *Maier et al. 2016* | --------- | net calcification | net calcification |
| *Swezey et al. 2017* | --------- | Proportion zooids Mg/Ca > 12 | Proportion zooids Mg/Ca > 12 |
| ***Growth*** |  |  |  |
| *Melzner et al. 2011* | length | length & somatic growth | somatic growth |
| *Hettinger et al. 2013* | shell growth | shell growth & total dry weight | total dry weight |
| *Pansch et al. 2014* | growth (mm) | growth (mm) & dry weight | dry weight |
| *Thomsen et al. 2013* | shell length | shell length & organic mass | organic mass |
| *Drenkard et al. 2013* | septa diameter | septa diameter & tissue lipid weight | tissue lipid weight |
| *Edmunds 2011* | Low temp as control | High temp as control (as specified in Edmunds 2011) | High temp as control (as specified in Edmunds 2011) |
| *Crook et al., Drenkard et al.*  *& Edmunds 2011* | Used SE incorrectly as SD, artificially reducing variability | Used correct value for SD | Used correct value for SD |
| *Oddvarsdotter 2014*  *(unpublished)* | carbon (ug C/L) | Not included, unpublished data | Not included,  unpublished data |
| *Taylor et al. 2014* | --------- | % change width included for LnRR effect of CO_2_ | Not included, ln (negative number) = NA |
| *Büscher et al. 2017* | --------- | growth (% per day) | growth (% per day) |
| *Cole et al. 2016* | --------- | shell length | shell length |
| *Hurst et al. 2017* | --------- | Larval growth (mm/day) and mass growth (mg/day) | Larval growth (mm/day) and mass growth (mg/day) |
| *Swezey et al. 2017* | --------- | growth efficiency | growth efficiency |
| ***All papers*** | Included intermediate food levels  No correction for multiple comparisons to control CO_2_ | Excluded intermediate food levels  Corrected for multiple comparisons to control CO_2_ | |

**Papers used for the main meta-analysis**

Büscher, J. V., Form, A. U. & Riebesell, U. Interactive effects of ocean acidification and warming on growth, fitness and survival of the cold-water coral *Lophelia pertusa* under different food availabilities. *Front. Mar. Sci.* **4,** 101 (2017).

Cole, V. J. *et al.* Effects of multiple climate change stressors: ocean acidification interacts with warming, hyposalinity, and low food supply on the larvae of the brooding flat oyster *Ostrea angasi*. *Mar. Biol.* **163,** 125 (2016).

Comeau, S., Carpenter, R. C. & Edmunds, P. J. Effects of feeding and light intensity on the response of the coral *Porites rus* to ocean acidification. *Mar. Biol.* **160,** 1127–1134 (2013).

Crook, E. D., Cooper, H., Potts, D. C., Lambert, T. & Paytan, A. Impacts of food availability and *p*CO_2_ on planulation, juvenile survival, and calcification of the azooxanthellate scleractinian coral *Balanophyllia elegans*. *Biogeosciences* **10,** 7599–7608 (2013).

Drenkard, E. J. *et al.* Calcification by juvenile corals under heterotrophy and elevated CO_2_. *Coral Reefs* **32,** 727–735 (2013).

Edmunds, P. J. Zooplanktivory ameliorates the effects of ocean acidification on the reef coral *Porites* spp. *Limnol. Oceanogr.* **56,** 2402–2410 (2011).

Hurst, T. P., Laurel, B. J., Hanneman, E., Haines, S. A. & Ottmar, M. L. Elevated CO_2_ does not exacerbate nutritional stress in larvae of a Pacific flatfish. *Fish. Oceanogr.* **26,** 336–349 (2017).

Hettinger, A. *et al.* The influence of food supply on the response of Olympia oyster larvae to ocean acidification. *Biogeosciences* **10,** 6629–6638 (2013).

Maier, C. *et al.* Effects of elevated *p*CO_2_ and feeding on net calcification and energy budget of the Mediterranean cold-water coral *Madrepora oculata*. *J. Exp. Biol.* **2,** jeb.127159 (2016).

Melzner, F. *et al.* Food supply and seawater *p*CO_2_ impact calcification and internal shell dissolution in the blue mussel *Mytilus edulis*. *PLoS One* **6,** (2011).

Pan, T.-C. F., Applebaum, S. L. & Manahan, D. T. Experimental ocean acidification alters the allocation of metabolic energy. *Proc. Natl. Acad. Sci.* **112,** 4696–4701 (2015).

Pansch, C., Schaub, I., Havenhand, J. N., Wahl, M. & Sciences, B. Habitat traits and food availability determine the response of marine invertebrates to ocean acidification. *Glob. Chang. Biol.* **20,** 765–777 (2014).

Ramajo, L. *et al.* Biomineralization changes with food supply confer juvenile scallops (*Argopecten* *purpuratus*) resistance to ocean acidification. *Glob. Chang. Biol.* n/a–n/a (2015). doi:10.1111/gcb.13179

Swezey, D. S. *et al.* Interactive effects of temperature, food and skeletal mineralogy mediate biological responses to ocean acidification in a widely distributed bryozoan. *Proc. R. Soc. B* **284,** 20162349 (2017).

Taylor, J. R. et al. Physiological effects of environmental acidification in the deep-sea urchin *Strongylocentrotus fragilis*. Biogeosciences 11, 1413–1423 (2014).

Thomsen, J. J., Casties, I., Pansch, C., Körtzinger, A. & Melzner, F. Food availability outweighs ocean acidification effects in juvenile *Mytilus edulis*: laboratory and field experiments. *Glob. Chang. Biol.* **19,** 1017–1027 (2013).

Towle, E. K., Enochs, I. C. & Langdon, C. Threatened Caribbean coral is able to mitigate the adverse effects of ocean acidification on calcification by increasing feeding rate. *PLoS One* **10,** 1–17 (2015).
